# Higher rates of fully preserved posterior cruciate ligament in total knee arthroplasty using a double tibial cut: a prospective randomized controlled trial

**DOI:** 10.1186/s43019-023-00208-z

**Published:** 2024-01-10

**Authors:** Gianluca Cinotti, Francesca Romana Ripani, Beatrice Perciballi, Giuseppe La Torre, Giuseppe Giannicola

**Affiliations:** 1 Department of Anatomical, Histological, Forensic Medicine and Orthopaedic Sciences, University La Sapienza, Piazzale Aldo Moro, Rome, Italy; 2 Aereospace Medicine Department, Italian Air Force, Rome, Italy; 3 Department of Public Health and Infectious Diseases, University La Sapienza, Piazzale Aldo Moro, Rome, Italy

**Keywords:** Total knee arthroplasty, Total knee replacement, Femoral roll back, Posterior cruciate ligament, Knee range of motion

## Abstract

**Purpose:**

In cruciate retaining total knee arthroplasty, posterior cruciate ligament damage may occur during tibial cutting. A prospective randomized study was conducted to investigate whether a novel tibial cutting technique was more effective than the currently used techniques.

**Materials and methods:**

Patients undergoing cruciate retaining total knee arthroplasty were recruited in a prospective, randomized, controlled trial. In 25 patients (group 1) the tibial cut was performed using a double tibial cut technique; in 25 (group 2) and 25 (group 3) patients, the bone island and en bloc resection techniques were performed, respectively. Posterior cruciate ligament integrity and femoral rollback were assessed at the end of surgery. The Oxford Knee Score, WOMAC score and range of motion were assessed postoperatively.

**Results:**

Posterior cruciate ligament was completely preserved in 92% of patients in group 1 and in 64% in group 2 and 3, respectively (*p* = 0.03). The Oxford Knee Score and WOMAC scores did not differ between groups (*p* = 0.4). The mean knee flexion was 126.4°, 121.5° and 123.9° in groups 1, 2 and 3, respectively (*p* = 0.04). The femoral rollback at 120° flexion was 80.7%, 72.2% and 75.4% in groups 1, 2 and 3, respectively (*p* = 0.01).

**Conclusions:**

The double cut technique preserves the posterior cruciate ligament at significantly higher rates than the bone island or en bloc resection techniques. Better posterior cruciate ligament preservation may improve the femoral rollback and knee flexion.

**Level of evidence:**

Prospective randomized controlled trial, Level I.

## Introduction

Clinical and experimental studies have shown the role of the posterior cruciate ligament (PCL) in the normal knee biomechanics [1–9]. The PCL, with its anterolateral and posteromedial bundles, is a primary constraint against the posterior translation force of the tibia during the range of motion (ROM) [1, 4, 5] and a secondary knee stabilizer under rotatory loading during flexion beyond 90° [4, 8]. As the function of the sagittal and rotatory stabilizers occurs synergistically with the posterolateral corner and medial ligament complex [4, 8, 9], overloading of these anatomical structures might occur when the PCL is injured.

The biomechanical functions of the PCL have been incorporated into the total knee arthroplasty (TKA) designs, including implants resembling the normal knee when the PCL is preserved and those with a post-cam mechanism when the PCL is sacrificed. Although both designs have provided high rates of satisfactory results, paradoxical or reduced femoral rollback, decreased tibial internal rotation, and changes in the patellofemoral contact area have been associated more frequently with the cruciate- retaining (CR) than with the posterior stabilized (PS) implants [2, 6, 7, 10–12]. Dynamic laxity and instability, which are associated with PCL insufficiency [13], are more difficult to detect and may explain why some patients remain unsatisfied after a well aligned CR TKA [14–16]. Nevertheless, an increase use of CR designs may be expected in the next years since several long-term investigations have shown greater survival of CR compared to PS TKA [17–19].

In CR TKA, the tibial cut may be performed en bloc or after the preservation of a bone island anterior to the PCL to protect the ligament insertion on the tibial facet. However, regardless of the technique used, there remains a substantial risk of damaging the PCL while making the tibial cut [20–26]. In a previous investigation analyzing the maximum thickness of a tibial cut that preserves the PCL, it was found that a 4–6 mm thick tibial resection could effectively preserve the PCL [20] and that a thinner cut should be performed in patients with a reduced posterior slope (< 5°), and vice versa, since a reduced posterior slope increases the risk of PCL division [20]. As most of the tibial components have a thickness of 9–10 mm, a second resection is needed. The second cut may be performed safely since, after the first cut, the trabecular bone in front of the PCL insertion is clearly visualized [20].

To our knowledge, no study has analyzed the effectiveness of different surgical techniques to preserve the PCL during tibial cutting. A prospective investigation was designed to evaluate the outcome of the PCL-sparing technique of double tibial cutting compared to currently used procedures, including the preservation of a bony island anterior to PCL insertion and en bloc resection technique. The hypothesis was that the double-cut technique could avoid PCL transection and preserve femoral roll back more consistently than the currently used techniques.

## Material and methods

### Patients

**Fig. 1:**
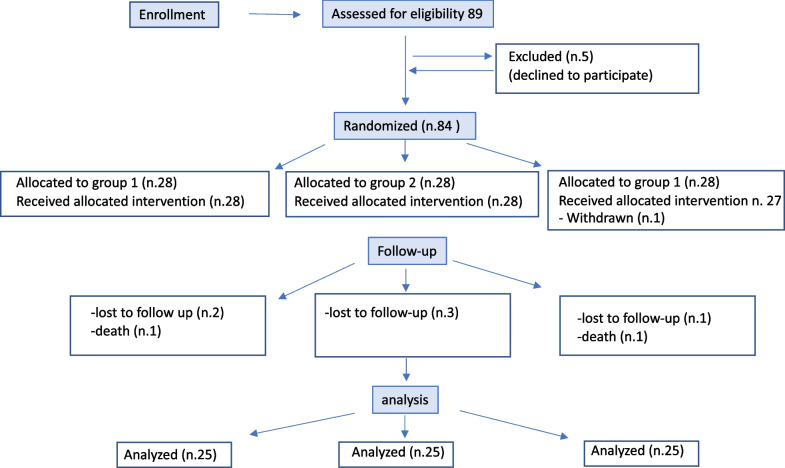
CONSORT (Consolidated Standards of Reporting Trials) flow diagram. Eighty-three patients were randomized in the study and 75 patients were analyzed

Patients scheduled for primary TKA between June 2017 and June 2019 were prospectively enrolled in a randomized controlled study designed to assess the effectiveness of a novel tibial cutting technique for PCL preservation. The inclusion criterion was primary or secondary knee osteoarthritis in which a CR TKA was indicated. The exclusion criteria were previous knee surgeries for degenerative or traumatic conditions; varus-valgus deformity greater than 15°, bone defects or severe flexion contractures (> 25°) requiring a PS implant; and patients unwilling to attend clinical follow-ups on a regular basis. Of the 88 patients enrolled in the study, five declined to participate; seven were lost to follow-up and one died at the 2-years follow-up. The remaining 75 patients, were included in the study (Fig. 1). A statistical calculator (EpiCalc2000 for Microsoft Windows, version 1.02) was used for the randomization process to generate a code that equally assigned each patient to one of the three groups of treatment, based on age, sex and priority for TKA. The latter included three classes based on the severity of pain, functional disability and Ahlbäck’s radiographic score [27]. The baseline demographic and clinical data are reported in Table 1.

**Table 1:** Baseline demographic and clinical data

Variables	Group 1 (25)	Group 2 (25)	Group 3 (25)	*p* value
Age	71.2 ± 8.4	72.3 ± 6.7	70.7 ± 6	n.s
Sex (Female/male)	15/10	16/9	16/9	n.s
BMI^§^ (kg/m^2^)	29.3 ± 4.2	28.6 ± 3.8	28.1 ± 4.6	n.s
Side (right/left)	16/9	14/11	13/12	n.s
OKS* (preoperative)	16.5 ± 5.8	17 ± 4.5	18.4 ± 4.1	n.s
Womac (preoperative)	64 ± 14	66.6 ± 11.5	64.3 ± 9.3	n.s
Varus/valgus deformity	19/6	21/4	20/5	n.s
HKA^ angle in varus knee	170 ± 8.1	168.9 ± 7.4	169.2 ± 7.5	n.s
HKA^ angle in valgus knee	187.2 ± 7.7	186.8 ± 8.3	187.6 ± 6.4	n.s
Preop. ROM**	107.5° ± 11.4	108.3° ± 13.8	109° ± 9.8	n.s

Mean values and (±) standard deviation in each group; ^§^ Body Mass Index;

^*^Oxford Knee Score; ^Hip-Knee-Ankle angle; **Range of motion

### Surgical treatment

**Fig. 2:**
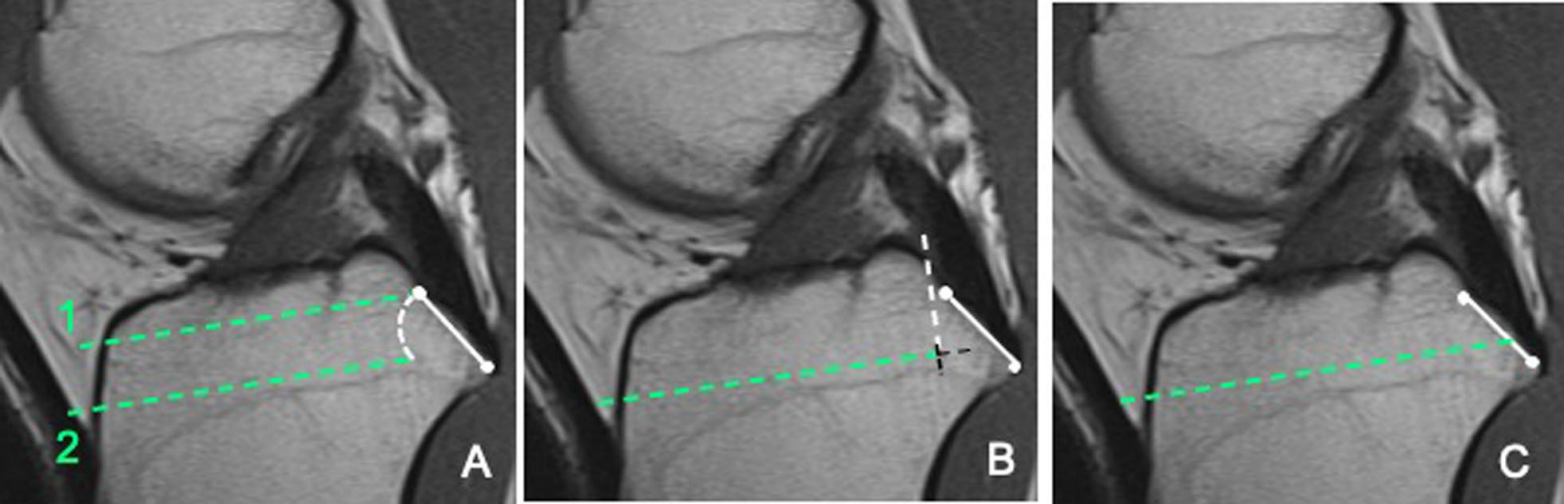
**S**agittal MRI scans showing the different techniques of tibial cutting. The white continuous line represents the PCL insertion in the PCL facet. **A** Double tibial cut technique. The dotted line 1 and 2 represent the first and second tibial cut; the white dotted line represents the residual bone adjacent to the PCL insertion after the second cut. **B** Bone island technique. The white dotted line represents a limited osteotomy performed to isolate a bone block in front of the PCL. When the bone block is inadvertently undermined by the saw blade (black dotted lines) its anchoring area is reduced leading to increased risks of detachment due to the traction exerted by the PCL. **C** en bloc resection. The image highlights the risk of damaging, and difficulty in protecting, the PCL insertion when this technique is used

All operations were performed under spinal anesthesia along with peripheral sciatic and femoral nerve blocks. A cemented TKA (Columbus, Aesculap) was implanted using a standard medial parapatellar approach by a senior surgeon (GC). In all patients a tourniquet was inflated before skin incision and deflated before arthrotomy closure. No patient underwent patellar resurfacing. The tibial extramedullary rod was aligned perpendicular to the anatomical axis in the coronal plane, with a posterior slope ranging from 3° to 7° (including 3° built in the polyethylene insert) depending on the native tibial slope. Anatomical references were used for coronal and sagittal alignments [20, 28]. To evaluate the effectiveness of different tibial resection modalities on PCL integrity, the tibial cut was performed using one of the three surgical techniques: double tibial cut technique (group 1), bone island preservation adjacent to the PCL insertion (group 2), and en bloc resection of the proximal tibia (group 3) (Fig. 2).

**Fig. 3:**
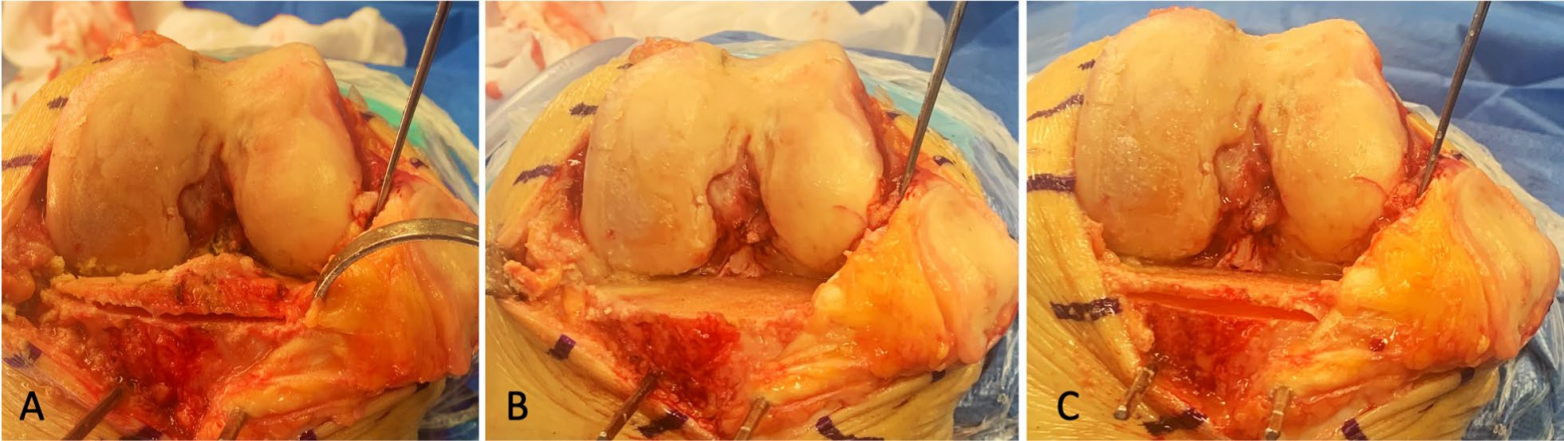
Intraoperative imaging of the double tibial cut technique. **A** Image showing the first tibial cut. **B** After the removal of the first slice of bone, the posterior tibial cortex adjacent to the PCL insertion is clearly exposed. **C**: Image showing the second tibial cut necessary to achieve the thickness of the tibial component

In the double tibial cut technique, an Hohmann’s retractor for PCL was used to protect the PCL fibers during the first tibial cut. The thickness of the first tibial cut varied according to the degree of the posterior tibial slope (PTS) of the operated knee and the posterior slope of the tibial cut [20, 24]. The PTS was calculated by averaging the posterior tibial slope of the lateral and medial tibial plateaus measured on lateral view radiographs. A 4 mm cut was performed in patients with PTS < 5°; a 5 and 6 mm thick tibial cut was performed in those with a PTS of 5°–8° and > 8°, respectively. Because the minimum thickness of the tibial component implanted was 10 mm, a second tibial cut was performed, after the first cut, to achieve a total thickness of 10 mm. As a result, when the first cut was 6 mm thick, the second cut was performed by shifting the cutting guide 4 mm caudally on the same pins; when the first cut was 4–5 mm thick, the second cut was performed by placing the cutting guide 5–6 mm below. In the latter, new pins were usually placed to lower the cutting guide of 5–6 mm. As during the second cut the trabecular bone in front of the PCL is clearly exposed, the saw blade is stopped before reaching the posterior tibial cortex leaving a few mm of trabecular bone in front of the PCL insertion (Fig. 3).

In group 2, a curved osteotome was used to isolate a round bone block in front of the PCL insertion. A distal ledge was placed 10 mm above its end to prevent cutting of trabecular bone beyond 10 mm and avoiding undermining of the bone island. In group 3 the tibial cut was performed en bloc having care to protect the PCL with an Hohmann’s retractor. In each group the tibial cut thickness was measured on the healthy side. The further surgical steps remained unchanged, including a coronal femoral cut aligned to the anatomical-mechanical alignment angle, rotation of the femoral component averaging the antero-posterior and posterior condylar axes and a rotation of the tibial component set at the medial third of the tibial tuberosity. In varus knees, ligamentous balancing was achieved by dissecting the deep medial collateral ligament (MCL) fibers. Subperiostal dissection of the superficial MCL was performed if the medial compartment remained too tight after the division of the deep fibers. In valgus knees, multiple puncture technique was used to release the lateral collateral ligament (LCL). The flexion space was first balanced with a femoro-tibial distractor to achieve a joint gap of about 1 and 2–3 mm in the medial and lateral side, respectively. The extension space was balanced to achieve 2° of varus-valgus laxity under manual stresses on the medial and lateral side.

PCL integrity was directly visualized and assessed using a probe by the operating surgeon along with a senjor and young resident. The PCL was graded as “preserved” when no macroscopic injuries or division of PCL fibers were seen and the ligament tensioning appeared normal. When part of PCL fibres were found to be cut and the tensioning of the ligament reduced it was graded as “partially recessed”. The PCL was graded as “fully divided”, when most or all fibers of the PCL appeared to be cut with an identifiable proximal and/or distal ligament stump. At the end of the surgery the tourniquet was removed and patellar tracking was assessed. Lateral retinacular release was performed if patellar tilt or dislocation was observed during flexion–extension of the knee. After skin closure the femoral rollback was assessed on the operating table under fluoroscopy by placing the knee at 90°and 120° of flexion using a digitalized goniometer.

The postoperative course included flexion–extension exercises and weight-bearing on the first postoperative day. The patient was discharged on the 3rd or 4th postoperative day. The rehabilitation program was continued for 4–6 weeks in the inpatient rehabilitation clinics.

### Clinical evaluation

Patients were evaluated before surgery, at 3, 6, and 12 months postoperatively, and up to a minimum of 2 years after surgery. The mean follow-up was 2.9 years (range 2.2–3.6 years). The clinical evaluation was performed by two senior residents who were not involved in the operation and were unaware of the study group assignments. The Oxford Knee Score (OKS) and Western Ontario and McMaster University Osteoarthritis Index (WOMAC) questionnaires were administered to all the patients. A digitalized goniometer was used to assess ROM and varus-valgus stability.

### Radiographic evaluation

**Fig. 4:**
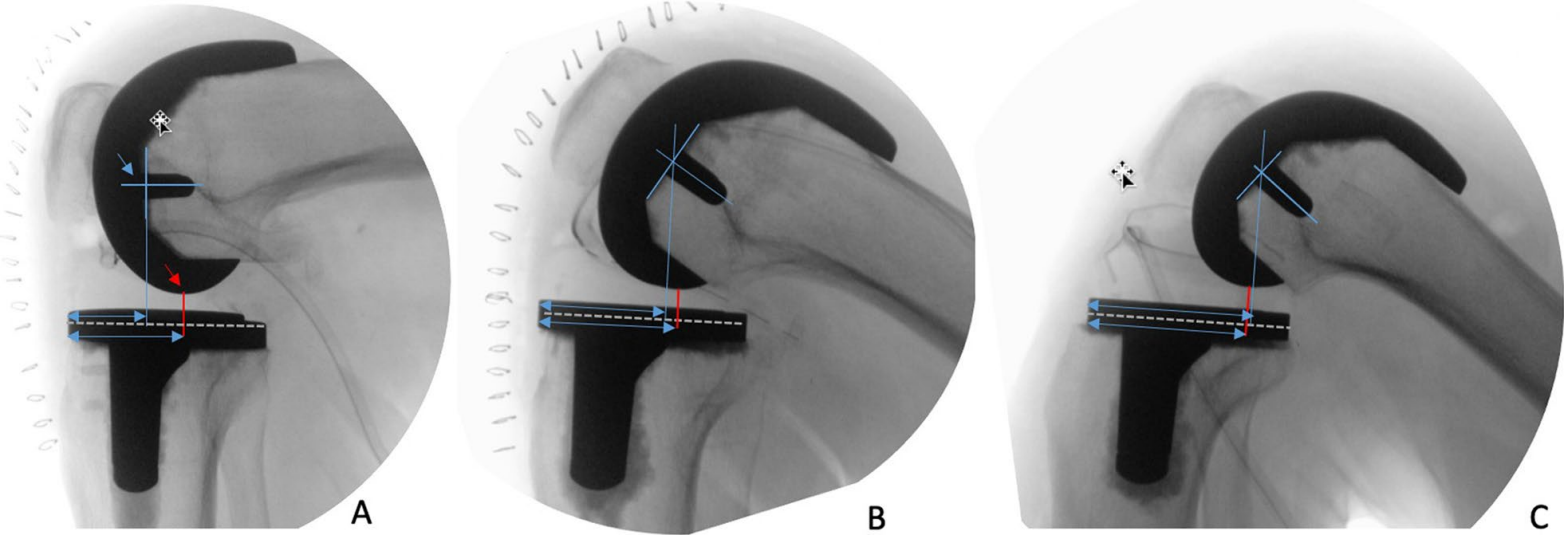
**A** Fluoroscopic images showing the reference points used to measure femoral rollback including the shortest distance between the femoral and tibial components (red arrow), and the femoral peg projection on the tibial component (blue arrow). **B** and **C** Show the relationship between the two reference points in patients with different degrees of femoral rollback

The hip-knee-ankle angle (HKA) and the implant alignment were measured. An HKA of 0° ± 3° of varus (−)/valgus (+) was considered to be within the normal range. The presence and progression of radiolucent lines or cement debonding were assessed using the Knee Society Evaluation System [29]. The femoral rollback was evaluated using fluoroscopic images taken at the end of surgery by two examiners who were blinded to the study groups. The degree of femoral translation was measured using two different references: (1) the shortest distance between the femoral and tibial components (SDFT) and (2) a line perpendicular to the tibial component passing through the base of the femoral component peg (the femoral peg projection [FPP]) (Fig. 4). To avoid the possible influence of different radiographic magnifications, the femoral rollback was expressed as a percentage of the sagittal diameter of the tibial component.

### Statistical analysis

The sample size was calculated based on the minimal clinically important difference of 15 points in the WOMAC score [30, 31]. A minimum sample size of 23 patients was necessary for each group with a power of 80% and an alpha error of 0.05. Considering a drop out rate of 3 to 5 patients per group (12–22%), 28 patients were recruited for each group. Fluoroscopic imaging was independently evaluated by 2 observers. The intraclass correlation coefficient (ICC) was calculated for intra- and inter-rater reliabilities. Intraobserver reliability was calculated by remeasuring the femoral rollback in 30 randomly selected images (10 per group) 2 months after the initial evaluation (10 per group). The Kolmogorov–Smirnov test was used to assess the normal distribution of the data. Independent t-test and repeated measures analysis of variance were used to estimate the difference in clinical scores between groups. Kruskall-Wallis one-way analysis of variance was used to assess differences in the ROM and femoral rollback between the groups. The chi-squared test was used to evaluate differences in the rates of PCL preservation between the groups. Statistical analyses were performed using SPSS for Windows (version 26.0; IBM Corp., Armonk, NY, USA).

## Results

The mean tourniquet time was 62.3 ± 8.6 (range 48–75), 61.7 ± SD 8.1 (range 47–77) and 59.6 ± 9.1 (range 46–78) minutes in group 1, 2 and 3, respectively, the difference being non significant (n.s.). Intraoperative assessment of PCL status before tibial cutting revealed that a competent PCL was present in all patients. After tibial resection, the PCL appeared to be divided in no patients in group 1 (0) and in five (20%) and two (8%) patients in groups 2 and 3, respectively (*p* = 0.03) (Table 2).

**Table 2:** PCL status after the tibial cut

PCL status	Group 1 (25)	Group 2 (25)	Group 3 (25)	p value
Preserved	23 (92%)	16 (64%)	16 (64%)	0.03
Partially divided	2 (8%)	4 (16%)	7 (28%)	
Entirely divided	0	5 (20%)	2 (8%)	

*p* = 0.03 between group 1 and 2; *p* = 0.04 between group 1 and 3; *p* = 0.3 between group 2 and 3

### Clinical results

The median preoperative OKS was 15 (range 9–28), 17 (range 9–28), and 18 (range 11–26) in groups 1, 2 and 3, respectively (n.s.). At the two-year follow-up, the median postoperative OKS was 42 (range 28–48; 95% CI 39–43.3), 41(range 26–48; 95% CI 37.4–41.7) and 41 (range 34–47; 95% CI 39.2–42.3), in groups 1, 2 and 3, respectively (n.s.). The difference between the pre- and post-operative OKS was 24.6 (range 19–37), 22.3 (range 13–35) and 22.4 (range 13–32), in groups 1, 2 and 3, respectively (*p* < 0.0001). The rate of improvement in the pre- and post-operative OKS did not differ significantly between the groups (n.s.) (Mean values are reported in Tables 1 and 3).

**Table 3:** Clinical outcomes and range of motion

Variables	Group 1 (25)	Group 2 (25)	Group 3 (25)	*p* value
OKS*				
3 months	33.7 ± 4.3	32.2 ± 5.3	32.4 ± 4.9	n.s.
12 months	37.8 ± 4.6	37 ± 4.7	37.6 ± 4.4	n.s.
2 years	41.2 ± 5.2	39.6 ± 5.2	40.8 ± 3.8	n.s
WOMAC				
3 months	34.2 ± 10	35 ± 8.4	36 ± 9.4	n.s.
12 months	23.8 ± 10.6	26 ± 8.8	26.4 ± 8	0.05^
2 years	18.8 ± 13.5	22 ± 8.8	23 ± 9.8	n.s.
ROM**	126.2° ± 4.7	121.5 ± 2.7	123.9 ± 3.1	0.0002^^^/0.03^§^/0.009^*^

^*^Oxford Knee Score; **Range of Motion. Difference (p value) between ^group 1 and 2; ^§^gr 1 and 3; *group 2 and 3. Mean values and standard deviations are reported

The median preoperative WOMAC scores were 67 (range 34–88), 69 (range 41–81) and 63 (range 51–79) in groups 1, 2 and 3, respectively (n.s.). At the two-year follow-up, the median postoperative WOMAC scores were 15 (range 1–48; 95% CI 13.2–24.3), 20 (range 8–42; 95% CI 18.3–25.6) and 23 (range 7–40; 95% CI 18.9–27) in groups 1, 2 and 3, respectively (n.s.). The WOMAC scores significantly improved postoperatively in all groups (*p* < 0.0001). However, the rate of improvement between the pre-operative and post-operative score was not different between the groups. The median preoperative pain score was 13 (6–17), 14 (8–17) and 13 (9–16) in groups 1, 2 and 3, respectively (n.s.). The median postoperative pain score was 2 (range 0–7), 3 (range 0–6) and 3 (range 0–5) in groups 1, 2 and 3, respectively (n.s.). The median preoperative function score was 48 (range 20–64) 47 (range 30–60) and 44 (range 34–54) in groups 1, 2 and 3, respectively (n.s.). The median postoperative function score was 14 (range 0–39), 17 (range (6–34) and 18 (range 4–34) in groups 1, 2 and 3, respectively (n.s.).

**Fig. 5:**
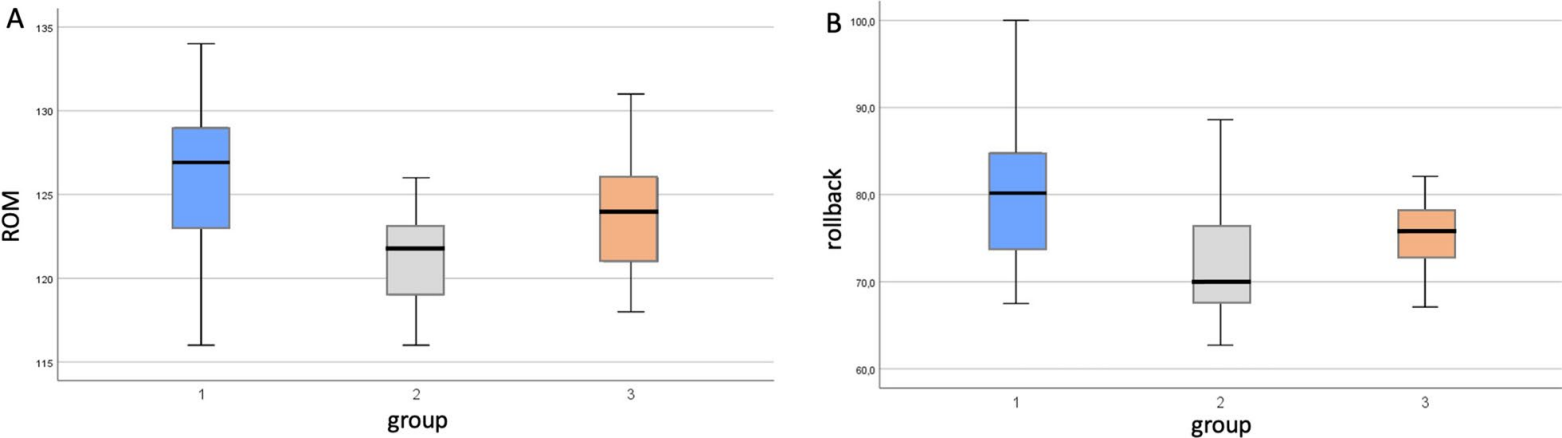
**A** Box plots showing the distribution of range of motion (ROM) and **B** the percentage of posterior femoral translation compared with the sagittal tibial plate diameter, in the 3 groups. The boxes represent the median and interquartile range (IQR). The errors bars represent the range of data

The median ROM was 126.2° (range 116°–134°; 95% CI 124.2–128.1), 121.5° (range 116°–126°; 95% CI 120.3–122.6) and 123.9° (range 118°–131°; 95% CI 122.6–125.1) in groups 1, 2 and 3, respectively (*p* = 0.0002) (Fig. 5a) (The mean values are reported in Table 3).

### Radiographic data

The radiographic results are shown in Table 4. The intra- and inter-observer reliability in evaluating the femoral rollback was 0.86 and 0.88, respectively. The median femoral rollback at 90° of flexion was, using the FPP reference, was 46.2% (range 32.8–59.6%; 95% CI 43.1–49.2), 41.8% (range 29.8–56.1%; 95% CI 39.1–44.4) and 43.3% (range 28.9–56.9%; 95% CI 40.4–46.1) in groups 1, 2 and 3, respectively. Using the SDFT reference, the median femoral rollback at 90° of flexion was 65% (range 49.3–76%; 95% CI 61.8–68.1), 59% (range 43.7–71.2%; 95% CI 56.2–61.7) and 62% (range 49.4% and 70.4%; 95%CI 60.1–63.9) in groups 1, 2 and 3, respectively. The difference was significant between groups 1 and 2 (Table 4). At 120° of flexion, the median femoral rollback (using the FPP reference) was 79.5% (range 62.5–94.7%; 95% CI 75.6–83.3), 70.9% (range 62.9–80.8%; 95% CI 68.6–73.1) and 74.1% (range 62.9–80.8%; 95% CI 71.7–76.4) in groups 1, 2 and 3, respectively (Fig. 5b). Using the SDFT, the median femoral rollback at 120° flexion was 80.7% (range 67.5–100%, 95% CI 77.1–84.2), 71.3% (range 60.9–85.7%; 95% CI 68.5–74) and 74.6% (range 60–2–85.1%; 95% CI 73–76.1) in groups 1, 2 and 3, respectively (Fig. 3b). The differences were significant between all groups (Table 4).

**Table 4:** Hip-Knee-Ankle (HKA) angle and femoral rollback at 90° and 120°

Variables	Group 1 (25)	Group 2 (25)	Group 3 (25)	*p* value
HKA angle	182.2° ± 3.4	181.7° ± 3.6	183.1° ± 2.9	n.s
Femoral rollback at 90°				
FPP**	46.2 ± 7.4	41.8 ± 6.5	43.3 ± 7	0.01^/ 0.07^§^/0.4^*^
SDFT^^	65 ± 7.7	59 ± 6.6	62 ± 4.6	0.006^/0.09^§^ /0.05^*^
Femoral rollback at 120°				
FPP	79.5 ± 9.3	70.9 ± 5.8	74.1 ± 5.6	0.0009^/0.01^§^/0.05^*^
SDFT	80.7 ± 8.5	71.3 ± 6.7	74.6 ± 3.8	0.0003^/0.01^§^/0.01^*^

^**^Femoral Peg Projection; ^^Shortest Distance between Femoral and Tibial components. Values of posterior femoral translation are expressed as % of the sagittal diameter of the tibial component Difference (*p* value) between ^group 1 and 2; ^§^ gr 1 and 3; ^*^ group 2 and 3

### Complications

A superficial wound infection was diagnosed in one knee in group 1 and 3. In both patients, intravenous antibiotics were administered for 2 weeks, when resolution of the superficial infection was seen. One patient in group 2 developed postoperative stiffness and required mobilization under general anesthesia. None of the patients required a reoperation.

## Discussion

The most important finding of the study was that the double tibial cut technique allows complete preservation of the PCL in a higher rate of patients than the bone island and the en bloc resection techniques and that a better ROM and greater femoral rollback are associated to higher rate of fully preserved PCL. The bone island technique does not provide better PCL preservation than the en bloc resection technique.

Current techniques for tibial cutting include the preservation of a bone island adjacent to the PCL or the en bloc resection of the proximal tibia [20, 29, 30, 32]. Both techniques carry the risk of inadvertently sacrificing the PCL. The preservation of a bone island anterior to the PCL insertion was introduced to protect the ligament insertion. However, during the tibial cutting the bone block may be undermined with the saw blade thus reducing its anchoring area on the underlying bone. As a result, during flexion, the traction forces exerted by the PCL may cause the detachment of the bone block along with PCL fibres [26] (Fig. 2). In the en bloc resection technique, a Hohmann’s retractor is used to protect the PCL insertion. However, the PCL fibers insert into the distal half of the PCL facet and it may be difficult to secure the ligament insertion in this area. In keeping with this, high rates of PCL division have been reported in imaging studies simulating the tibial cut [22–24] in TKA and in cadaveric and clinical investigations analyzing PCL fibers removed with the excision of the tibial plateaus [21, 25, 26, 33].

As current CR TKA techniques do not seem to guarantee the complete preservation of the PCL during tibial cutting and, to the best of our knowledge, no previous study has explored possible alternatives, a prospective study was designed to assess the effectiveness of a novel PCL-preserving technique. The rationale for the double tibial technique originates from an imaging study showing that, to fully preserve the PCL, a tibial cut should be 4 to 6 mm thick to end above the insertion of the PCL. As the PCL fibers run close to the posterior cortex before inserting into the distal half of the PCL facet [34], they are at risk of being cut and must, therefore, be protected with a retractor. The technique requires a second cut to achieve a total thickness of 9–10 mm for tibial component implantation. In this study, the double tibial cut technique was compared with two currently used techniques: en bloc resection and preservation of a bone bloc anterior to the PCL insertion. The posterior slope of the tibial cut ranged between 0 and 4°, which led to an overall posterior slope between 3° and 7° considering that a liner with a 3° of posterior slope was used. The PCL was completely preserved in 92% of the patients in the study group compared to 64% of patients in the bone island and en bloc resection groups. None of the patients in the double tibial cut group had entirely divided PCL while this occurred in five (20%) and two (8%) patients in the bone island and en block resection groups, respectively. The postoperative clinical scores significantly improved in all groups. The OKS did not differ significantly between the groups. A better WOMAC score was found in the study group than in group 2 at the 1- year follow up but results were similar at the 2 years follow-up.

Several investigations have analyzed femoral rollback in patients undergoing TKA using fluoroscopic imaging [35–37]. However, most of previous studies included a limited number of patients in whom different types of TKA were compared [35–37]. Kim et al., analysed the femoral rollback using intraoperative sensors in patients who had a CR and PS TKA [38]. The authors found that PS design provided significant better femoral rollback during flexion up to 120° compared to CR. In this study the femoral rollback was greater in the group of the double cut technique than in the other groups; it was associated with greater ROM but did not influence the clinical outcome. In patients undergoing the double-cut technique femoral rollback was similar to that found in patients with PS TKA [38], meaning that when the PCL is preserved femoral rollback may be similar with both CR and PS designs.

In this study the bone island technique did not perform better than the en bloc resection technique. Although the rate of complete PCL preservation was similar in both groups, a greater proportion of patients in the bone island group showed complete PCL division than those in the en bloc resection group (20% versus 8%). Slightly better ROM was measured in the en block resection group than in the bone island group, although the difference was not significant. An in vitro investigation comparing the two techniques showed that PCL damage occurred in 23% and in 73% of patients in the bone island and en bloc resection groups, respectively [33]. However, the authors did not strictly simulate the tibial cut performed in TKA; a 2-mm thick tibial cut was made from the medial tibial plateaus which, in a healthy knee, leads to an excessively thin tibial cut [33].

The clinical relevance of a PCL recession or division during tibial cutting in CR TKA is yet to be established [30, 39–44]. Dion et al. retrospectively analyzed a series of patients undergoing TKA in whom the PCL was retained, recessed or fully divided [30]. The latter was accomplished in the presence of excessive femoral rollback and anterior lift-off of the tibial trial. The clinical and functional scores did not differ significantly among the three groups; the authors concluded that it is not necessary to convert a CR to PS when the PCL had been recessed or excised [30]. However, the authors analyzed the effect of PCL recession or division in patients in whom such a release was performed due to an excessively tight flexion space while they did not analyze patients in whom the PCL division, though not necessary, occurred inadvertently. In contrast, in the present study, patients in whom preoperative knee stiffness and /or deformity suggested that a PS knee could be advisable were excluded from the study. Furthermore, with the trial component in place, no knee was judged too tight to require a PCL release or division. Although patients were informed that a PS could be an option in case of unbalanced flexion space, a PS was not implanted in cases showing a full division of the PCL, because a balanced flexion space was still achieved in these cases.

This study has certain limitations. The accuracy of the macroscopic assessment of PCL integrity during surgery is not known since we did not perform a postoperative MRI to confirm the intraoperative findings. In addition, the fact that the operating surgeons were aware of the surgical techniques used may have biased the intraoperative evaluation of the PCL integrity after the tibial cut. However, the intraoperative evaluation was in line with the postoperative assessment of femoral rollback and ROM which were blindly evaluated. It should be also considered that when few PCL fibers are divided and the ligament is evaluated as fully preserved, the ligament function is likely to be preserved and vice versa. Second, whether the assessment of femoral rollback on the operating table under spinal anesthesia is representative of femoral rollback in vivo may be debated. However, femoral rollback can be affected by pain so it may be more difficult to compare the degree of femoral translation at definite flexion angles in vivo. Third, as femoral rollback may be affected by the degree of tensioning or laxity of the flexion space and by ligament balancing, they may influence femoral translation. Although this possible source of bias cannot be excluded, a priori, a technique aimed at obtaining a balanced flexion space was adopted in all patients. On the other hand, in no patient in this series was there a narrow flexion space such that a PCL release would required. Fourth, although a power analysis was conducted prior to the study, a larger sample size may reveal possible differences in the clinical scores which may be concealed by other factors not identified in this study. Nevertheless, this is the first study analyzing the results of several tibial cutting procedures on PCL preservation in TKA. Fifth, as the liner of the tibial component used includes 3° of the posterior slope the results of this study do not necessarily apply to other TKA without a posterior slopped liner.

## Conclusions

Current tibial cutting techniques in TKA are associated with a risk of PCL damage. Although the extension of PCL fibers resected during surgery may vary in relation to the native inclination of the tibial plateaus and the posterior inclination of the tibial cut, a surgical technique aimed at preserving the PCL more consistently would be advisable. This prospective randomized study has shown that the double cut technique preserves the posterior cruciate ligament at significantly higher rates than the bone island or en bloc resection techniques. Better posterior cruciate ligament preservation may improve the femoral rollback and knee flexion.

## Data Availability

The datasets generated and/or analysed during the current study are available from the corresponding author on a reasonable request.
